# Creasote

**Published:** 1860-10

**Authors:** 


### ARTICLE VI.

### *Creasote.*

This is the most valuable remedy of the whole materia medica to the dentist, filling a want not to be so well supplied by any other agent. We therefore accept of it as a subject of the present paper, and may be excused for entering into some of the minor details of its history, characteristics and uses. Since its discovery by Reichenbach, it has had but few additional facts added to the good uses established for it by him, principally owing no doubt to the offensive and extremely persistent odor attendant upon its presence. It is obtained from almost all organic substances by destructive distillation, principally however from common tar, pyroligenous acid, wood smoke and some oils, being a laborious process, besides excessively disagreeable. The agents used, according to Turner, are potash, phosphoric and sulphuric acids, with ammonia.

Thus by frequent distillations creasote is eventually freed from its oily affinities, presenting a colorless and transparent fluid, much heavier than water, and boiling at 397°F., preserving its fluidity at 16.6°F. It absorbs chlorine by becoming resinified, a fact useful to know in the process of bleaching discolored dentine, which we think should be treated by this agent after the various chlorides have been used, so that any altered tissues may receive a preservative consistence.

Creasote decomposes nitric acid and is itself decomposed by potassium. It is soluble in alcohol, ether, sulphuret of carbon, euphion, naptha, acetic acid, (which makes a powerful caustic,) and acetic ether. One of its very valuable qualities to the dentist, is its capacity to hold resins in solution, which forms one of the best applications to exposed and inflamed nerves, suspending pain and at the same time affording a temporary shield against foreign matters.

A singular fact exists in its purely medicinal use, which renders it a difficult drug to administer, it forms but two combinations with water, 1.25 parts of creasote to 100 of water, the other, 10 parts of water in 100 of creasote.

One of the most powerful antiseptics with all animal tissues, it is again most efficient to the dentist in its application to carious teeth, whose offensive decomposition is more than corrected by it, for the power it has of coagulating all albuminous solutions, and also of changing the action of decomposition into more or less of an unchanging one, is especially useful, at the same time it is harmless upon the fibrous and earthy tissues. It is seldom to be had pure, as many substances are mixed with it, the only simple practical test however is its colorless quality, and even this is no safeguard against its adulterations with some oils, &c. Ettling gives the following analysis of creasote, which has been generally regarded as correct:

| | | | |
|---|---|---|---|
| Carbon, | 14 | Eq. wt. 84 | per ct. 77.42 |
| Hydrogen, | 9 | " " 9 | " " 8.12 |
| Oxygen, | 2 | " " 16 | " " 14.46 |
| Creasote, | 1 | 100 | 100.00 |

Pereira says, however, the equivalent of creasote must be considered as uncertain.

Its effect upon vegetable life is a uniform slow death, no doubt by attacking the organic oils and resinous constituents, from its strong affinity thereto. Upon animals, it has a no less startling result than hydrocyanic acid, destroying insects, as spiders, flies, small fishes, in a few moments, by subjecting them to water containing but a few drops. Injected into the veins of larger animals, it suspends the action of the heart, hurries respiration, and followed by convulsions and death. When injected into the stomach it is attended by disturbed vision, as dimness or fixedness of sight, vertigo and coma.

Upon man, the last author quoted says, creasote operates locally as an irritant and caustic; applied to the skin it causes heat, redness, and destruction of the cuticle. On the tongue it produces a painful sensation. In contact with a suppurating surface it acts similarly to lunar caustic.

When taken in large doses it excites vomiting and purging, attended by a sense of burning and rapid alternations of great heat, especially in the pharynx, œsophagus, stomach and head, with vertigo and head-ache.

A singular feature of its exhibition is noticed by Dr. Elliottson, who knew a lady who increased the dose of creasote to forty drops before it disagreed; the addition of a single drop beyond this produced its usual symptoms of excess.

Its principal use in medical practice is to suppress vomiting, and has been found most effectual in cases of excessive nausea from pregnancy.

Perhaps its very best use is for dental purposes, as for instance in the inflammation of the periosteum of a tooth, it seems to be the only remedy at all efficacious. It is applied by saturating a soft cotton string and tying it around the tooth in contact with the gum; when this can be done it will generally be found to give relief, and if properly pursued by frequent and careful applications, will eventually subdue most cases of periostitis.

In the destruction of a nerve of a tooth it should always constitute a part of the application, to subdue the pain and irritation that arsenic will give rise to. A paste is commonly made of equal parts of morphia and arsenic with creasote, and thus brought in contact with the pulp it will most frequently produce the death of this part with but little if any pain. After the pulp has been removed, the tooth should be treated with this agent for some days, to purify the parts and induce a healthy cicatrization of the severed ends of the nerve and blood vessels. In teeth that have died from whatever cause, they should before filling, be subjected to a free use of creasote, for experience proves that this agent has a preservative power in this operation, which however difficult to explain, must be received as a fact, as well as a power to prevent subsequent inflammation, which to the dentist is an indispensable and invaluable quality.

We have not found it serviceable in obviating that extreme sensibility dentine so often assumes, and which is perhaps the most formidable difficulty of practice, not even in any degree, and the impossibility of making it continuously applicable in such cases, prevents the experiment being continued for a length of time except in some few cases of large cavities; even in these cases when an opportunity from the shape of the cavity has offered, other circumstances have prevented the test, so that we have not been induced to enumerate it as among the remedies for the reduction of the sensibility of dentine.

For tooth-ache, it certainly acts as a specific, when skillfully applied, for much depends upon the manner any application is made to an aching tooth. The difficulties are generally found in the neglect of two important cares, first, the absolute removal of all loose or foreign matters, or entire cleanliness, and the compression of air upon the nerve by covering the surface of the cavity with the wax before pressing it firmly, in order that it will be effectually retained. When a cavity is properly prepared in an aching tooth, and the saturated lint partially filling the cavity applied, the wax covering should be perforated with a hole so as to allow the free egress of air, otherwise the condensation of the air upon the inflamed pulp will be a sufficient cause for the continuance of the pain, but when properly done it will invariably mitigate the pain, if not entirely subdue it.

We mentioned the value of a solution of resin in creasote for this purpose, greatly aiding the exclusion of foreign matters, by forming a perceptible coat to the tender part, which fills the office of camphor effectually, as proposed by our books, in connection with this agent.

The successful treatment of abscess of the gums also imperatively demands creasote, injected into them if possible, if not, its application within their orifices as far as possible. Dr. Ballard, of New York, proposed inserting delicate pieces of lint saturated with it, renewing the same until healthy granulation appeared or cicatrization followed. This is the only treatment for dental abscess that can be termed at all successful.

We have used it most satisfactorily in disease of the antrum, where we found injections of diluted nitrate of silver, 4 grains to the oz., iodide of iron, &c., produce but little good effect, when creasote used 4 to 10 drops to the oz., acted with great success in relieving the pain and checking the discharge. One case of eight years standing, proved an entire cure under its use, inside of three weeks treatment.

In epulis we have used it in connection with lunar caustic with great satisfaction. Although it is by no means an active cautery, yet it acts by slow measure to the destruction of a part by a painless process which places it ofttimes as superior to more potential agents. One case in particular of this disease, we treated with the most immediate and happy result. It had been forming for about eighteen months, until it had reached the size of a large pea. I applied potassa opt. by placing a piece upon the tumor, and confined it there by wax formed around it and the neighboring teeth, occasionally using a little creasote to relieve the pain arising from the rapid decomposition of the tumor; in the course of four hours it was entirely removed, and the part freely treated with creasote, when in a few days a healthy cicatrice formed, proving entirely successful, without the common resort to the knife.

Indeed as a cautery, few substances will equal this preparation of potassa, and for this purpose, we deem it most valuable, because its action is much less injurious to natural teeth, especially when many have been filled, when nitrate of silver, or preparations of iron would induce bad consequences.

The writer's experience with it has been limited, but so far results have justified the highest estimation of it as a potent cautery. The experiments now being tried, as an application to destroy the nerve of a tooth, are not quite so satisfactory as could be desired, but this may be in a great measure attributable to the imperfect knowledge of its use, but a more extended experience is demanded to establish its efficacy in this particular.

Returning from this digression, we lastly come to notice creasote as a remedy for excessive ptyalism.

Burnt alum, various preparations of soda, vegetable astringents, and many washes, have all proved abortive in correcting that peculiar condition of the mouth resulting from mercurialization. But a wash of creasote, diluted at first to a great degree, and increased gradually up to twenty drops to the ounce, will be found to correct this unfortunate state more speedily and effectually than any other known remedy.

We translate from a French journal a few brief statements in connection with its general use. A Dr. Hasbach states that he has cured cases of cancrum oris with creasote, the disease being developed in children living in low, damp, and otherwise unhealthy places. It is spread upon the diseased parts with a hair pencil, the gangrenous parts readily separating. M. Arendt employs it for uterine hemorrhages, by injecting a solution in proportion two drops of creasote to five ounces of water. Also in bleeding from the nose. For external applications its strength should be increased from ten to twenty drops to the ounce, and applied by saturating bandages.

For burns it is said to relieve the pain, but not thought generally applicable. Dr. Forsten advises it in all erectile tumors, also in erysipelas. For diseases of the skin it is widely known as a sovereign remedy, and constitutes a chief feature in all palliative remedies for ulcers and cancerous tumors.

